# IgM nephropathy complicated by cerebral venous sinus thrombosis: a case study

**DOI:** 10.1186/s12882-020-02048-5

**Published:** 2020-09-07

**Authors:** Elizabeth Downie, Jason Diep, Nagendraprasad Sungala, Jeffrey Wong

**Affiliations:** 1 Department of Renal Medicine, Liverpool Hospital, Sydney, NSW Australia; 2 Department of Haematology, Liverpool Hospital, Sydney, NSW Australia

**Keywords:** IgM nephropathy, Cerebral venous sinus thrombosis, Thrombosis, Nephrotic syndrome

## Abstract

**Background:**

IgM nephropathy is a rare disease with variable clinical presentations and is an unusual cause of nephrotic syndrome. Histopathological findings typically include mesangial hypercellularity with IgM and complement deposition, though the spectrum may range from normal glomeruli through to focal and segmental glomerulosclerosis. Thromboembolism is a well recognised complication of nephrotic syndrome, but cerebral venous sinus thrombosis is rarely described.

**Case presentation:**

This is the case of a 23-year-old male presenting with the nephrotic syndrome, whose initial renal biopsy was consistent with minimal change disease. Complete remission was achieved with prednisone, however multiple relapses and steroid dependence prompted re-biopsy, the results of which were more consistent with IgM nephropathy. His last relapse was complicated by cerebral venous sinus thrombosis. He then received rituximab and a weaning course of prednisone to again enter remission.

**Conclusions:**

This case highlights the need to consider IgM nephropathy in the differential diagnosis of nephrotic syndrome. Additionally, it emphasises the risk of thrombosis in patients with severe nephrosis.

## Learning points

- Always consider thromboses as a cause of unusual symptoms when patients have severe nephrosis
- Have a low threshold for repeating a renal biopsy if the clinical picture is not consistent with the initial histological diagnosis
- IgM nephropathy is a rare condition with variable presentation, ranging from microscopic haematuria through to the nephrotic syndrome

## Background

Immunoglobulin M nephropathy (IgMN) is a rare disease with variable presentation, from microscopic haematuria through to the nephrotic syndrome (NS) [1]. A recent large case series showed IgMN to have a prevalence of 1.8% of all native kidney biopsies [2]. The typical findings of IgMN on light microscopy (LM) include mesangial hypercellularity which may be mild, with immunofluorescence (IF) showing IgM as the sole or dominant immunoglobulin in the mesangium of the glomeruli in a diffuse and global pattern [3]. Associated with the immunoglobulin M (IgM) deposition is complement deposition, specifically complement component 3 (C3) [4]. However, the histological findings can vary from no glomerular abnormalities through to mesangial hyperplasia associated with segmental and global sclerosis [5]. This variability has led to ongoing conjecture about how IgMN fits into the spectrum of glomerulonephritis (GN) [1]. This case demonstrates the importance of re-biopsy and considering IgMN in the differential diagnosis of steroid dependent nephrotic syndrome in a young man thought to have Minimal Change Disease (MCD).

Thromboses are a common complication of nephrotic syndrome [6]. The risk of thrombosis increases with certain histopathological diagnoses (namely membranous nephropathy), increasing age, hypoalbuminaemia and recent diagnosis (within the last 6 months) [7]. The pathogenesis of thrombosis is related to increased loss of antithrombotic factors by the kidneys in conjunction with increased production of prothrombtic factors by the liver [6]. The most common locations of thrombosis include the deep veins of the legs, pulmonary arteries (as emboli) and renal vein [8]. This case study demonstrates the importance of considering thromboses in other locations when patients have severe nephrosis.

## Case presentation

A 23-year-old man presented to hospital with a 2-week history of oedema, frothy urine and lower abdominal pain. This is on a background of ulcerative colitis (UC) for which he was on sulfasalazine. He denied any other regular medications, over the counter medications or herbal supplements. His family history included his mother having IgA nephropathy. He was an occasional smoker and rarely consumed alcohol. He denied illicit drug use. On examination, he was mildly tachycardic (105 beats per minute) and normotensive. He was 180 cm tall and weighed 93 kg. He had mild bilateral pitting oedema to his knees. The rest of his examination was unremarkable. Investigations revealed normal renal function with a creatinine of 75 μmol/L, hypoalbuminaemia with a serum albumin of 15 g/l and urinalysis revealing 3+ protein. His urinary albumin to creatinine ratio (uACR) was 944.9 mg/mmol. His total cholesterol was 8.3 mmol/L. GN, vasculitis, infection and myeloma screen (dsDNA, C3 & C4, anti-PLA-2R, hepatitis B & C serology, serum free light chains and serum protein electrophoresis with immunofixation) were negative, with the exception of a positive atypical perinuclear anti-neutrophil cytoplasmic antibody (p-ANCA). Importantly the myeloperoxidase (MPO) and proteinase 3 (PR3) titres were both < 1 IU/ml. ANCA positivity is most likely explained by our patient’s history of UC where ANCA positivity is reported in 60–80% of patients, with a predominant p-ANCA pattern [9].

During preparation for renal biopsy our patient was found to have a prolonged activated partial thromboplastin time (aPTT) of 47 s. This corrected on a mixing study. Factor 12 deficiency (29%) was subsequently identified. Haematology opinion was that this conferred no increase in in vivo bleeding risk.

**Fig. 1:**
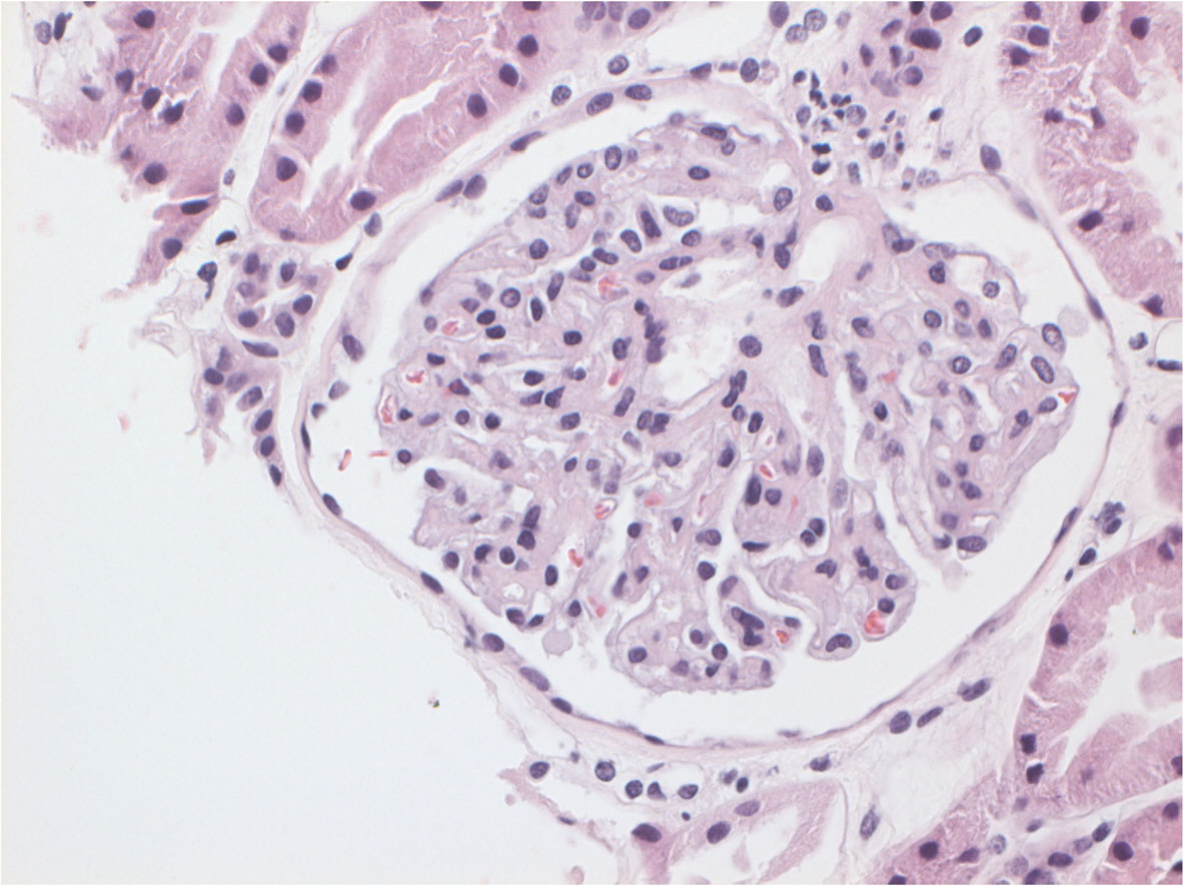
No morphological abnormality seen on light microscopy using haematoxylin and eosin staining

Renal biopsy was undertaken with LM revealing no morphological abnormality (see Fig. 1). IF revealed nonspecific trace glomerular deposits of IgM and C3. MCD was the most likely diagnosis. Our patient was consequently commenced on 75 mg oral prednisone daily.

After 1 month our patient achieved complete remission. He was no longer oedematous, his serum albumin was 33 g/l (from 15 g/L) and uACR was 0.7 mg/mmol (from 944.9 mg/mmol). His prednisone was slowly weaned from 75 mg daily to 50 mg daily for a fortnight and then 25 mg daily for a fortnight. Our patient remained in remission with a serum albumin of 39 g/L, total cholesterol of 6 mmol/L and no albuminuria.

**Fig. 2:**
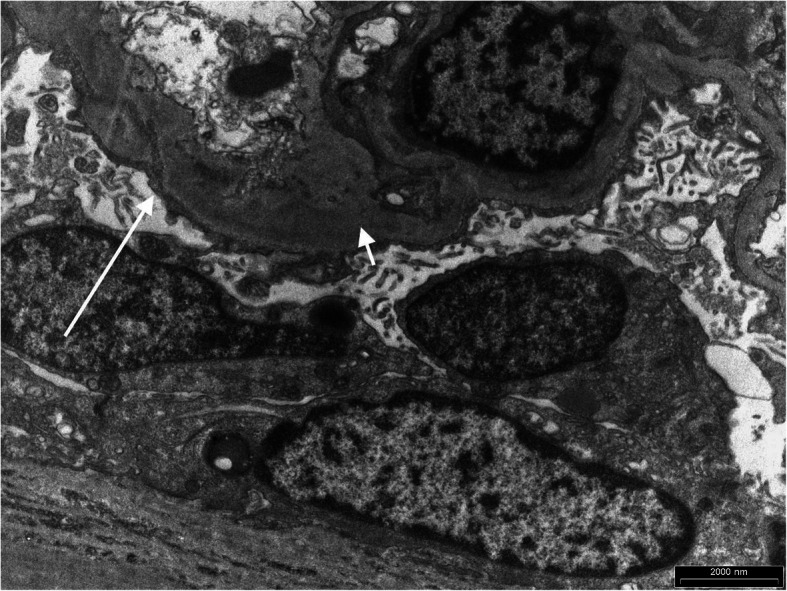
The delayed electron microscopy image showing mesangial expansion with deposits (short arrow) and foot process effacement (long arrow)

The electron microscopy (EM) report from our patient’s renal biopsy returned 2 months later. This revealed patchy foot process effacement, and mesangial expansion with some deposits suggestive of a mesangiopathic process (see Fig. 2).

**Fig. 3:**
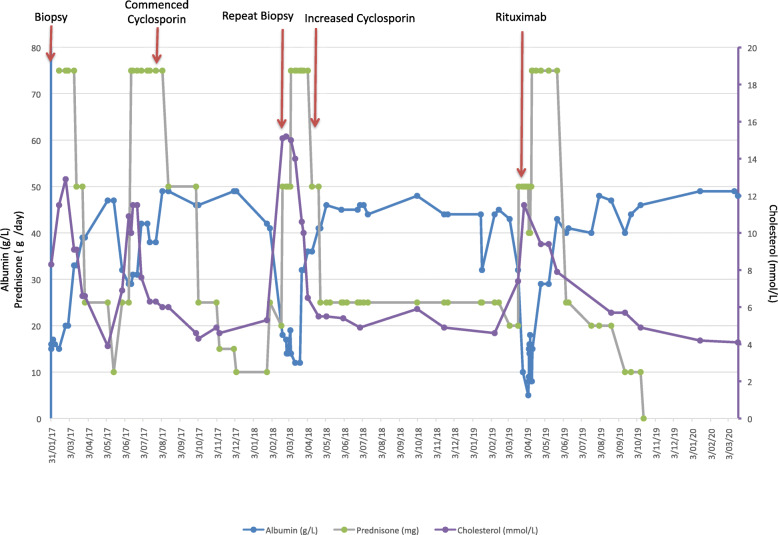
Our patient’s serum albumin, serum cholesterol and pharmacological treatments including prednisone, cyclosporin and rituximab over the course of his illness

Our patient then suffered a dramatic relapse with the prednisone dose at 20 mg/day. He reported recurrence of frothy urine, 4 kg of weight gain and proteinuria (on urine dipstick given for home monitoring). His prednisone was increased to 25 mg daily but despite this his oedema worsened and proteinuria persisted. Therefore his prednisone dose was further increased to 75 mg daily to induce remission (see Fig. 3).

A complete clinical remission was achieved over the next 2 months with serum albumin improving to 38 g/L and total cholesterol to 6.3 mmol/L. At this stage he was commenced on oral cyclosporin 100 mg BD as a steroid sparing agent given the previous failed attempt to wean steroids. Over the next 5 months our patient’s prednisone dosage was weaned more slowly. At a dose of 10 mg prednisone daily he suffered a second relapse with recurrence of oedema in association with albuminuria and hypoalbuminaemia to his previous levels. His prednisone dose was increased back to 50 mg daily and then 75 mg daily to re-induce remission.

**Fig. 4:**
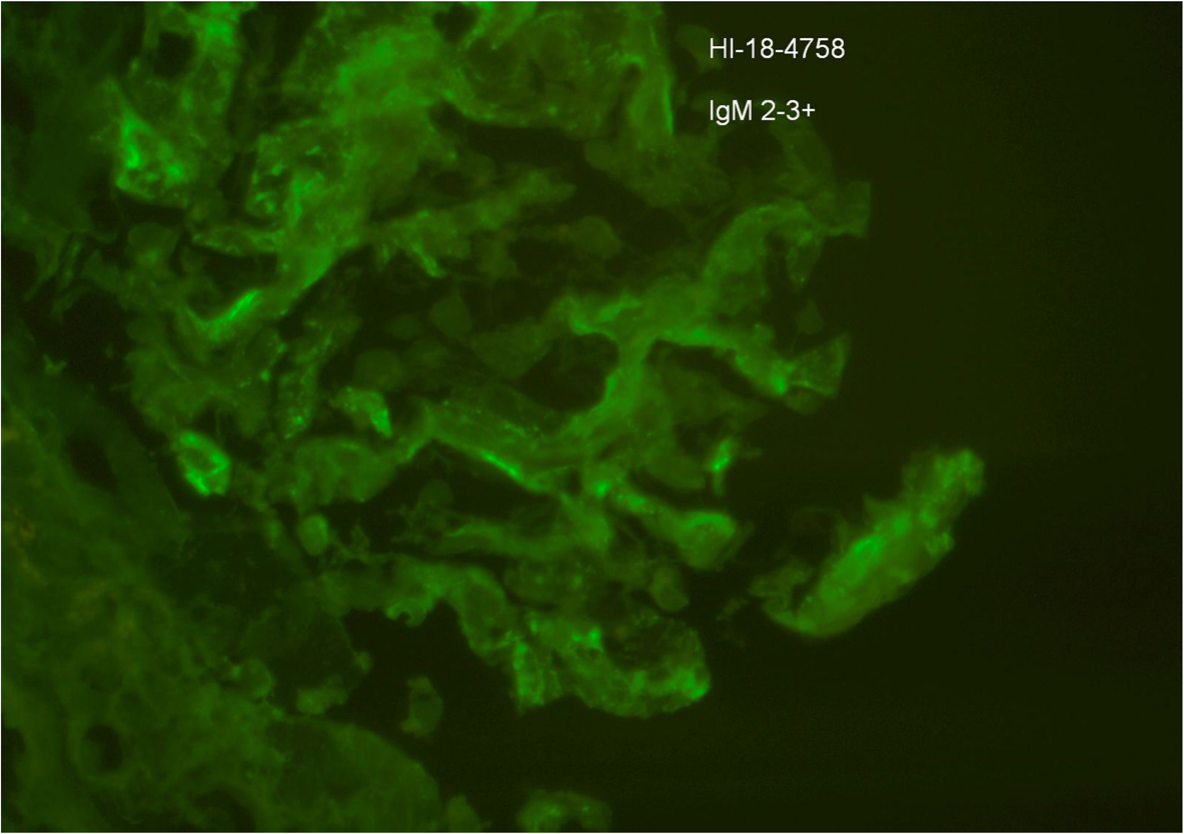
Immunofluorescence showing 2–3+ mesangial staining for IgM

**Fig. 5:**
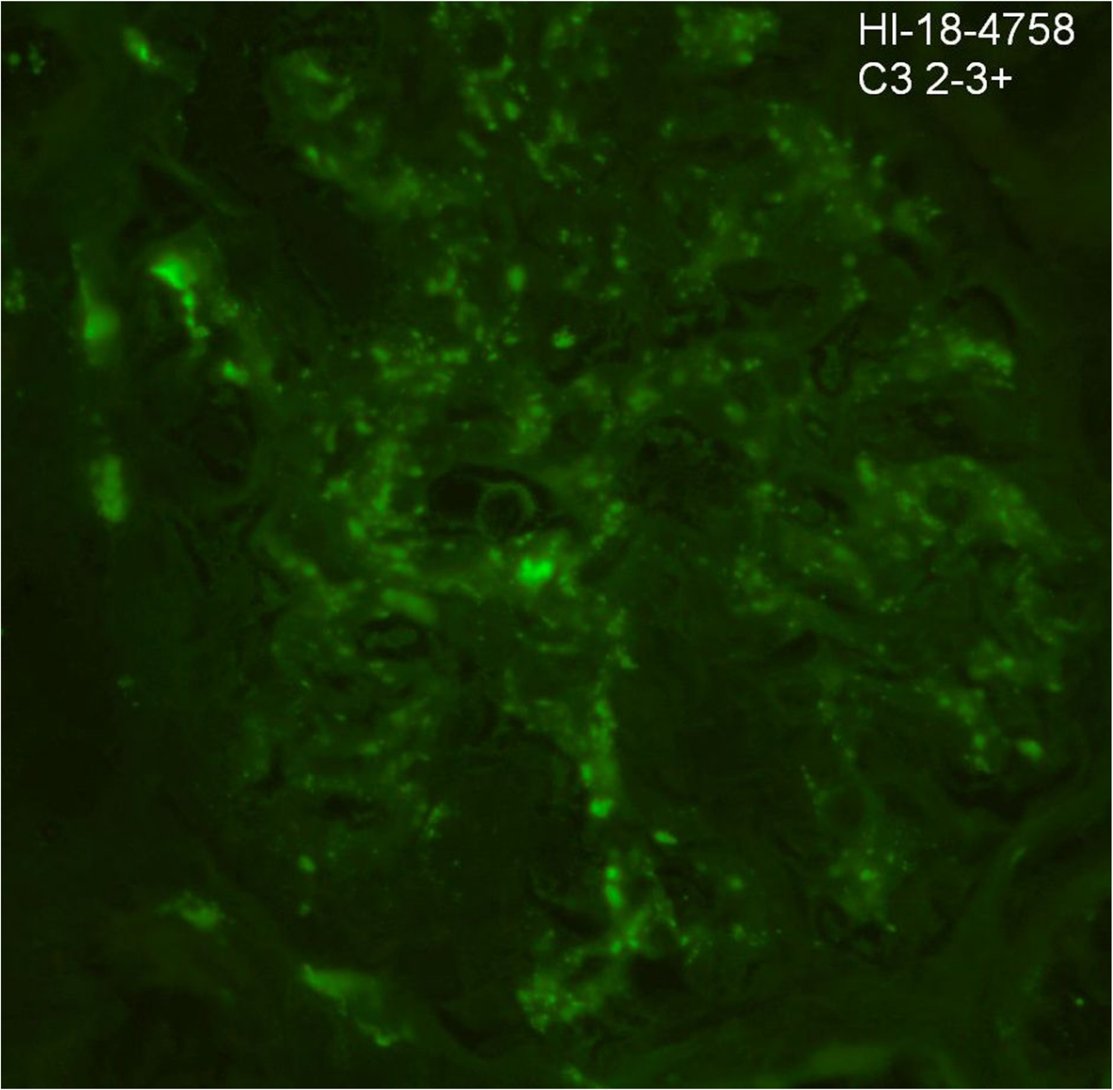
Immunofluorescence showing 2–3+ mesangial staining for C3

Given his multiple relapses, steroid dependence and initial EM report suggestive of a mesangiopathic process, our patient underwent a second renal biopsy. LM was again normal, however IF found significant mesangial deposits of IgM (2–3+) and C3 (2–3+) (see Figs. 4 and 5). IgM nephropathy was then consider a possible diagnosis.

This time our patient took longer to achieve remission, requiring 75 mg prednisone daily and an increase in his cyclosporin dose to 125 mg BD. His prednisone dose was again weaned to 25 mg daily. He also self-ceased his cyclosporin. Our patient was adherent to the recommended prednisone dose as he recognised that without it he would relapse, and he felt further relapses would impact his career progression.

After more than 12 months on prednisone 25 mg daily, and a total of 2 years on continuous corticosteroids, our patient agreed to a slow wean of the corticosteroid dose. Our plan was to use anti-human CD20 (Rituximab) if severe nephrosis recurred. He once again relapsed as soon as his prednisone dose reached 20 mg daily. At this stage his prednisone was increased to 50 mg daily, and he received the first of two planned doses of Rituximab 1 g as an outpatient. He suffered no adverse reactions from the rituximab. During this relapse, his serum albumin reached 10 g/L, 24 h urinary protein was 14.76 g/day and his total cholesterol was 11.5 mmol/L (see Fig. 3).

Several days after the rituximab infusion, our patient presented to the Emergency Department with a severe headache, nausea and vomiting. His headache had been progressively worsening since the day prior to his rituximab infusion. There were no focal neurological signs on examination. Computed tomography brain (CTB) and computed tomography angiogram (CTA) revealed a right transverse sinus thrombosis and non-occlusive posterior sagittal sinus thrombosis. We found no evidence of any inherited or acquired thrombophilia with extensive testing (anti-thrombin 3, factor V Leiden, lupus anticoagulant, prothrombin gene mutation, protein C & S, JAK 2 mutation analysis and paroxysmal nocturnal haemoglobinuria assay). He was commenced on therapeutic enoxaparin and bridged to warfarin.

He went on to receive a second dose of rituximab 1-month later. Since then he has remained in complete remission (see Fig. 3). His prednisone was weaned over the following 6 months, and his prednisone was ceased. A repeat CTB and CTA showed resolution of the transverse sinus and posterior sagittal sinus thromboses and a decision was made to stop anti-coagulation.

## Discussions and conclusions

### Discussion

This is a case of a young man with NS initially diagnosed with MCD, who underwent repeat biopsy as he did not respond as expected to treatment. His repeat biopsy was more consistent with IgMN. This case was complicated by the development of a cerebral venous sinus thrombosis (CVST). Venous thromboembolic events are a common complication in nephrotic syndrome, however cerebral venous sinus is an uncommon location.

The main weakness of this case study is that it is a single case describing a rare condition. Strengths include the several years of follow-up with our patient, as well as repeated investigations including renal biopsy, serum albumin, serum cholesterol and proteinuria.

IgMN was first described by 2 separate groups in the 1970s [4, 10], but debate has persisted as to whether this is its own disease or whether it represents a subset of FSGS or MCD. IgMN has a broad spectrum of clinical presentations ranging from microscopic haematuria to NS [1]. IgMN tends to affect children and young people, with a mean age of 29 and with 33% of patients being < 16 years in a Finnish study of 110 patients [5].

In our patient the initial biopsy was normal on LM. IF revealed only trace staining of IgM and C3. Literature suggests that trace IgM staining is not unexpected in MCD [5]. Based on this our patient was diagnosed with MCD. He went on to relapse firstly on prednisone alone and then again on cyclosporin and prednisone. His corticosteroid dependence and the EM suggestive of a mesangiopathic process made us doubtful of the initial diagnosis of MCD. His second biopsy was more consistent with typical findings of IgMN with a mild proliferation in mesangium on LM and 3+ IgM and C3 deposition on IF.

In the past the first line treatment for IgMN has been corticosteroids, though many patients are steroid resistant. In the Finnish study, 28% of adult patients were resistant to corticosteroids, and close to 36% were dependent on corticosteroids [5]. This is in contrast to MCD which is steroid sensitive in 85–90% of cases [11]. Previously second line treatment was with oral cyclophosphamide [5]. More recently, there have been several case studies showing successful treatment with rituximab in native [12] and transplant kidneys [13]. As of yet there are no randomised controlled trials focused on rituximab and its dosing as a treatment option. The two case studies using rituximab for IgMN used 375 mg/m^2^ 4-weekly for 2 doses [13] or 1 g 4-weekly for 2 doses [12]. Betties & Roodnat [13] demonstrated depletion of circulating CD20-positive B cells to less than 0.01 × 10^9^ CD20-positive cells/L with just 2 doses of rituximab. This, in addition to our unit’s familiarity with the 1 g IV rituximab fortnightly for 2 doses, lead to our decision to follow this dosing regimen. Our patient responded well to rituximab and has now been weaned off prednisone.

The occurrence of CVST in patients with NS has been described previously in case reports, particularly in the paediatric population. The mechanism of thrombosis in NS involves urinary losses of plasminogen, anti-thrombin 3, protein S and protein C, as well as increased platelet aggregation, increased viscosity of blood and defects in the fibrinolytic system [14].

Part of the difficulty in diagnosing CVST is the sometimes vague and non-specific symptoms. Additionally, CVST is uncommon, with an incidence up to 1.57 per 100,000 [15]. In adults with NS the most common locations for thrombosis are in the deep veins of the lower limb, pulmonary artery (as embolism) and renal vein [8]. CVST are less commonly reported in adults with NS, however have been reported in children with NS [6]. Headache is the most common symptom of CVST, occurring in 89% of patients [16]. Other presentations include focal neurological deficits, seizures and encephalopathy with mental state change [17]. The headache of CVST often has a slow onset over days as seen in our case [16].

The mainstay of treatment for CVST is therapeutic anticoagulation, in our case in the form of enoxaparin 100 mg BD subcutaneously and then warfarin orally (aiming for target INR 2–3) [18]. Despite having other risk factors for thromboembolic disease including factor 12 deficiency and ulcerative colitis, the haematology team advised that the final flare of his NS was the greatest contributing factor to our patient’s thromboses. Interestingly, one paper suggested that factor 12 deficiency specifically can occur in conjunction with NS as the small molecular weight of factor 12 makes it prone to increased urinary losses [19]. Our patient’s therapeutic anticoagulation was ceased after imaging proven resolution of his CVST. He has now been 6-months off therapeutic anticoagulation with no recurrence of symptoms.

Our patient initially presented with significant oedema and feeling generally unwell. At this time, and with each relapse, he described withdrawing socially as well as from sporting interests. He completely stopped playing rugby union for several years. He achieved excellent remission with corticosteroids and, over 12 months later, can still describe the minimum dose of 20 mg daily at which he would relapse. The frequent relapses lead to his reluctance to wean the corticosteroid dose. Our patient has no issues with rituximab, having no infusion reactions and suffering no infections. He is following-up with his nephrologist soon to discuss ongoing doses of rituximab.

### Conclusion

IgM nephropathy is a condition that should be considered as a differential diagnosis to MCD when the patient does not respond as expected to corticosteroid therapy. Additionally in all patients with NS, there should be a low threshold for consideration of thromboses in a diverse range of locations.

## Data Availability

Not applicable

## Abbreviations

LM: Light microscopy
IF: Immunofluorescence
IgMN: Immunoglobulin M nephropathy
IgM: Immunoglobulin M
C3: Complement component 3
GN: Glomerulonephritis
FSGS: Focal segmental glomerulosclerosis
MCD: Minimal change disease
UC: Ulcerative colitis
IgA: Immunoglobulin A
uACR: Urinary albumin to creatinine ratio
p-ANCA: Perinuclear antineutrophil cytoplasmic antibody
MPO: Myeloperoxidase
PR3: Proteinase 3
APTT: Activated partial thromboplastin time
EM: Electron Microscopy
CTB: Computed Tomography Brain
CTA: Computed tomography angiography
NS: Nephrotic syndrome
CVST: Cerebral venous sinus thrombosis
